# Effects of Nystatin oral rinse on oral *Candida species* and *Streptococcus mutans* among healthy adults

**DOI:** 10.1007/s00784-023-04969-5

**Published:** 2023-03-24

**Authors:** Mohammed Aljaffary, Hoonji Jang, Nora Alomeir, Yan Zeng, Naemah Alkhars, Shruti Vasani, Abdullah Almulhim, Tong Tong Wu, Sally Quataert, Jennifer Bruno, Aaron Lee, Jin Xiao

**Affiliations:** 1 Eastman Institute for Oral Health, University of Rochester Medical Center, Rochester, NY USA; 2 Imam Abdulrahman Bin Faisal University, Dammam, Saudi Arabia; 3 Temple University, Philadelphia, PA USA; 4 Faculty of Dentistry, Department of General Dental Practice, Kuwait University, Kuwait City, Kuwait; 5 Biostatistics and Computational Biology, University of Rochester Medical Center, Rochester, NY USA; 6 Immunology and Microbiology, University of Rochester Medical Center, Rochester, NY USA; 7 Perinatal Oral Health, Eastman Institute for Oral Health, University of Rochester, 625 Elmwood Ave, Rochester, 14620 USA

**Keywords:** *Candida albicans*, *Streptococcus mutans*, Nystatin, Salivary cytokines, Caries

## Abstract

**Objectives:**

To examine the effect of Nystatin oral rinse on oral *Candida species* and *Streptococcus mutans* carriage.

**Materials and methods:**

Twenty healthy adults with oral candidiasis participated in the single-arm clinical trial and received Nystatin oral rinse for 7 days, 4 applications/day, and 600,000 International Units/application. Demographic-socioeconomic-oral-medical conditions were obtained. Salivary and plaque *Candida species* and *Streptococcus mutans* were assessed at baseline and 1-week and 3-month follow-ups. Twenty-four salivary cytokines were assessed. *Candida albicans* isolates underwent Nystatin susceptibility test.

**Results:**

Half of participants (10/20) were free of salivary *C. albicans* after using Nystatin rinse. Salivary *S. mutans* was significantly reduced at 3-month follow-up (*p* < 0.05). Periodontal status reflected by bleeding-on-probing was significantly improved at 1-week and 3-month follow-ups (*p* < 0.05). Plaque accumulation was significantly reduced at 1-week follow-up (*p* < 0.05). Interestingly, the responses to Nystatin oral rinse were not associated with race, gender, age, oral hygiene practice, adherence to Nystatin rinse, or sweet consumption (*p* > 0.05). No *C. albicans* isolates were resistant to Nystatin. Furthermore, salivary cytokine eotaxin and fractalkine were significantly reduced at 3-month follow-up among participants who responded to Nystatin rinse (*p* < 0.05).

**Conclusions:**

The study results indicate that oral antifungal treatment had an effect on *S. mutans* salivary carriage. Future clinical trials are warranted to comprehensively assess the impact of antifungal treatment on the oral flora other than *S. mutans* and *Candida*.

**Clinical relevance:**

Due to the potential cariogenic role of oral *Candida* species, antifungal approaches shed new light on the prevention and management of dental caries from a fungal perspective.

**Supplementary information:**

The online version contains supplementary material available at 10.1007/s00784-023-04969-5.

## Introduction

Oral candidiasis is the most common fungal infection, with overgrowing *Candida* species in the superficial epithelium of the oral mucosa [1]. Among the oral *Candida* species, *Candida albicans* is the most prevalent fungal species that can cause oral candidiasis. Other *Candida* species often detected less frequently in the oral cavity include *C. tropicalis*, *C. guilliermondii*, *C. glabrata*, *C. parapsilosis*, *C. dubliniensis*, and* C krusei* [2]. *C. albicans* and oral bacteria have well-documented symbiotic relationships in oral mucositis, periodontal diseases, implant-related infections, and oral cancer [3]. Polymicrobial interactions between *C. albicans* and oral microbes affect the biofilm’s cellular and biochemical composition, which influences clinically relevant outcomes of biofilm-related oral diseases such as pathogenesis, virulence, and drug resistance [3, 4]. The interactions between *C. albicans* and coexisting oral bacteria occur through physical attachment, extracellular signals, and metabolic cross-feeding [3, 5].

Dental caries is a chronic infectious disease defined as the local destruction of dental hard tissues by acidic by-products from bacterial fermentation of dietary carbohydrates [6]. While *Streptococcus mutans* and *Lactobacillus species* have traditionally been considered the prime microbial risk markers and preventive targets for dental caries [7], recent research on the role of *Candida species* in caries and its synergistic interaction with *S. mutans* has shed new light on potential fungus-focused approaches to early prediction and subsequent prevention of dental caries.

The presence of *Candida* species in the oral cavity is usually found to be positively correlated with poor oral hygiene and high carbohydrate intake [8]. The following scientific evidence supports its potential cariogenic role: (a)* Candida* species (especially *C. albicans*) have often been detected at higher levels in the oral cavity of children with caries, compared to caries-free children [7, 9–11], and are positively correlated with caries severity [12]. A recent meta-analysis showed that children with oral *C. albicans* had 5-time greater odds of experiencing caries than children without this yeast strain. Similar findings have been reported among the adult population in our recent study [13]. (b) Our cross-sectional study [12] reported that the oral *C. albicans* carriage is also positively correlated with *S. mutans* carriage and a more cariogenic oral microbiota among individuals with caries. (c) Laboratory findings have added plausible biological evidence of the cariogenic traits of *C. albicans*. *C. albicans* is (a) acidogenic and aciduric [14, 15]; (b) capable of dissolving the major tooth component, hydroxyapatite, at an approximately 20-fold rate higher than *S. mutans* [16]; (c) capable of increasing *S. mutans* cells in biofilms through a unique *C. albicans*-*S. mutans* adhesive interaction [17, 18] that is mediated by extracellular polysaccharides (EPS) formation [17–19]; (d) enhancing the virulence of *S. mutans* when grown in *S. mutans*-*C. albicans* duo-species biofilm setting, with upregulated expression of genes that are related to microbial metabolism and cariogenicity, such as *gtfB*, *gtfC*, and *gtfD* [20]; and (e) capable of enhancing virulence and causing more severe caries when co-infected with *S. mutans* in a rodent model [17].

Due to the potential cariogenic role of oral *Candida* species and the synergistic interaction between oral *Candida* and cariogenic bacteria *S. mutans*, a hypothetic regimen to prevent and treat dental caries would be reducing the carriage of oral *Candida* by administering an antifungal medication and subsequently reduce the cariogenic interaction between *Candida* and cariogenic bacteria.

A recent study assessed the effect of fluconazole and povidone-iodine (PI) on inhibiting *C. albicans* and cariogenic bacteria in vitro and in an animal model [21]. This study indicated that a combination of fluconazole and PI has a moderate killing effect against *S. mutans* and lead to oral microbiota changes in rats [21]. In addition, the study revealed that added PI might enhance the antifungal activity of fluconazole while disrupting biofilm exopolysaccharide and *S. mutans* microcolony formation, reducing the bulk and density of infection in the animal model.

However, fluconazole is a systemic antifungal medication, and has not been routinely prescribed to treat oral candidiasis. Instead, Nystatin is considered the first-line medication for treating uncomplicated oral candidiasis [1]. Each 1 mL of Nystatin oral suspension contains 100,000 International Units (IU) of Nystatin and the following components: sodium carboxymethylcellulose, methyl p-hydroxybenzoate (E218), propyl p-hydroxybenzoate (E216), sodium metabisulphite (E223), sucrose, saccharin sodium, sodium citrate, permaseal aniseed flavor, and purified water [22]. The recommended dose of Nystatin ranges from 100,000 to 200,000 IU, four times/day for newborns/infants, and 200,000 to 600,000 IU, four times/day for children and adults [23].

Intriguingly, our recent study has shown that Nystatin application altered the formation and characteristics of *C. albicans* and *S. mutans* duo-species biofilms in an in vitro setting [24]. Even though Nystatin is the most commonly used antifungal medication to treat oral candidiasis, no studies have assessed the effect of Nystatin oral application on the carriage of *S. mutans* and other oral microorganisms in vivo. Therefore, the purpose of this clinical trial was to evaluate the effect of oral application of Nystatin suspension on oral microorganisms, including the fungal and bacterial communities, with an emphasis on cariogenic bacteria. The study results could lead to a better understanding of whether the oral antifungal treatment affects the carriage of oral cariogenic microorganisms.

## Materials and methods

### Study design

A single-arm non-randomized clinical trial was conducted among twenty study participants who met the inclusion and exclusion criteria at the Eastman Institute for Oral Health, University of Rochester. The study protocol was approved by the University of Rochester Research Subject Review Board (#STUDY00004638). This study is registered at the Trials.gov (#NCT04550546). This study followed the Strengthening the Reporting of Observational Studies in Epidemiology (STROBE) reporting guideline.

### Participants

Study participants were recruited from the existing pool of patients at the University of Rochester Medical Center (URMC) Eastman Institute for Oral Health (EIOH) clinics and from the larger community at Rochester NY.

The inclusion criteria include (a) 18 years and older; (b) positive oral *Candida* detection with sufficient oral *Candida* burden to meet the laboratory criteria for a diagnosis of oral candidiasis, ≥ 400 colony forming unit (CFU)/mL of salivary *Candida* [25]; and (c) ≥ 10,000 CFU/mL of *S. mutans* in the saliva.

The exclusion criteria include (a) visible signs of candidiasis on the mucosa or tongue at screening; (b) with systemic diseases, such as HIV, cancer, or diabetes; (c) history of using local (oral) or systemic antibiotics or antifungal medication within the last 3 months; and (d) women who are currently pregnant or reported that she is currently breast feeding. A pregnancy test (urine test) was conducted to exclude participants who are pregnant; (e) with more than 8 missing teeth (third molars and orthodontically extracted teeth are not included); (f) with more than 4 decayed teeth; (g) with removable dental prosthesis that are used to restore missing teeth; and (h) allergy to Nystatin.

### Study procedures

Study participants who entered the trial were instructed to rinse the mouth with (6 ml of 100,000 IU/mL) Nystatin suspension, without swallowing the suspension, at the frequency of four times per day (every 6 h) for 1 week. The Investigational Drug Services dispensed the Nystatin oral suspension at the University of Rochester Medical Center. The Nystatin suspension bottle was returned to quantify the unused quantity of Nystatin and assess the adherence to measure. In addition, a treatment adherence log was filled out by the study participants.

### Study flow

**Fig. 1:**
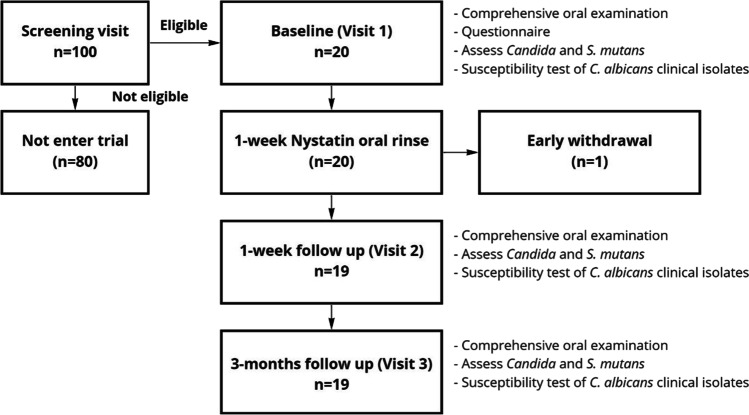
Study flow chart. All eligible study participants were enrolled and received a 1-week supply of Nystatin oral suspension at baseline visit. Study participants were followed at two additional visits post antifungal treatment

The study flow is demonstrated in Fig. 1. The study participants were assessed at 3 time points: (1) baseline visit (V1), (2) 1 week after the completion of Nystatin oral rinse (V2), and (3) 3 months after the completion of Nystatin oral rinse (V3).

### Data collection and examination

At the baseline visit, data on demographic and socioeconomic background were collected using a questionnaire. Data on the medical background and medications were self-reported and confirmed by electronic medical records. The medical background included (1) physician-diagnosed systemic diseases (Y/N), such as hypertension, diabetes, asthma, anxiety, depression, and kidney disease; (2) medications that subjects were taking at the baseline study visit; and (3) smoking status (Y/N). The oral hygiene practice was collected using a questionnaire.

The comprehensive examination was performed at the baseline and each study visit by a dentist in a dedicated examination room at the URMC, using standard dental examination equipment, materials, and supplies. Caries was scored using DMFT (decayed, missing, and filled teeth).

### Periodontal parameters

Bleeding on probing (BOP) was evaluated to assess the gingival inflammation. The periodontal tissue was assessed to the bottom of the clinical pocket or sulcus with a periodontal probe. Interproximal sites for every existing tooth, except the third molars, were scored from the buccal and the lingual sides [26]. Dental plaque accumulation was assessed using the Plaque Index (PI) described by Löe [27]. Each of the four gingival areas of the tooth was given a score of 0–3. A score of 0 indicates no plaque in the gingival area; a score of 1 indicates the presence of a film of plaque adhering to the free gingival margin; a score of 2 indicates moderate accumulation of soft deposits within the gingival pocket, on the gingival margin, and or adjacent tooth; and a score of 3 indicates an abundance of soft matter within the gingival pocket and or on the gingival margin and adjacent tooth structure.

### Saliva and plaque sample collection

Methods used for saliva and plaque sample collection were detailed previously [12, 13]. Approximately 2 ml of whole non-stimulated saliva samples were collected by spitting into a sterile 50 ml centrifuge tube. Study subjects were instructed not to eat, drink, or brush their teeth 2 h before the oral sample collection prior to their study visit. Supragingival plaques from the whole dentition were collected using a sterilized periodontal scaler. The plaque samples were suspended in 1 ml of a 0.9% sodium chloride solution in a sterilized Eppendorf tube.

### Identification and quantification of *Candida spp. and S. mutans*

The clinical samples (saliva and plaque) were stored on ice and transferred to the lab located at the Center for Oral Biology, UR, within 2 h for laboratory testing. The saliva and plaque sample were gently vortexed and sonicated to break down the aggregated plaque before plating. The sonication cycle was repeated three times, with 10-s sonication and 30-s rest on ice. BBL™ CHROMagar™ Candida (BD, Sparks, MD, USA) was used to isolate and identify *C. albicans*. *S. mutans* was isolated using Mitis Salivarius with Bacitracin selective medium and identified by colony morphology [28, 29]. Both *Candida spp.* and *S. mutans* were incubated at 37 °C, 5% CO2, for 48 h before identification. Colony PCR was used for a further identification of those *Candida spp.* and *S. mutans* that were unable to be identified by colony morphology [30]. The CFU values of *Candida spp.* and *S. mutans* on each plate were recorded.

### Susceptibility of *C. albicans* isolated to Nystatin in vitro

Antifungal susceptibility testing was performed according to the Clinical and Laboratory Standards Institute (CLSI) M27-A3 broth microdilution method using (RPMI 1640; Gibco) growth medium (with glutamine and without bicarbonate). The medium was buffered with 3-(N-morpholino) propanesulfonic acid (MOPS: Fisher Scientific) at a pH of 7.0 and to a final concentration of 0.165 mol/l. Clinical isolated *C. albicans* were recovered from frozen stock using Yeast Peptone Dextrose Agar (YPD) and incubated overnight at 37 °C with 5% CO2. The antifungal agent tested was Nystatin (VWR International, Sanborn, NY, USA). A stock drug was prepared (100 times the higher concentration) and aliquots in small volumes for future use. Two-fold serial dilutions were prepared, yielding final concentrations ranging from 0.06 to 32 ug/ml. The yeast inoculums were prepared by picking up young colonies of appropriate size from the 24-h old fresh cultures and suspending them in 5 mL of RPMI 1640 medium. The resulting suspension cell density was adjusted to 0.5 McFarland standard, corresponding to a stock inoculum of 1–5 × 10^6^ CFU/ml. Alternatively, the cells were counted using a hemocytometer. The suspension was diluted twice: first 1:20 and then 1:50 with RPMI medium to obtain a final working density of 1–5 × 10^3^ CFU/ml. Subsequently, 100 ul of this working inoculum solution and 100 ul of antifungal solution (at a working concentration twice higher than the desired final concentration) were seeded into a sterile, disposable, flat-bottomed flat 96-well plate (Greiner Bio-One). In each well, the final inoculum concentration was 0.5–2.5 × 10^3^ CFU/ml. The 96-well plates were incubated in ambient air without agitation at 35 °C and read spectrophotometrically at 600 nm after 24 h and 48 h using a 96-well microtiter plate reader (infinite M200 PRO, Tecan). In the case of the spectrophotometer readings, the MIC50 and MIC90 were determined. *C. albicans* SC 5314 was used as a reference for quality control in all the experiments.

Susceptibility to Nystatin was assessed using the MIC of each isolate. The isolates were classified as susceptible (S), susceptible dose-dependent (SDD), and resistant (R) and expressed visually as heatmaps. The interpretive criteria used were according to CLSI guidelines: Nystatin: resistant ≥ 16 ug/ml.

### Salivary cytokine level

Cytokine/chemokine assessment for 24 analytes was performed in the University of Rochester Human Immunology Center Core Lab facility on saliva samples collected at 4 time points (baseline, 1 week, and 3 months after Nystatin rinse). Samples were centrifuged for 5 min at 125,000 rcf at 4 °C prior to incubation of the sample at neat concentration overnight at 4 °C in two multiplexed magnetic bead array assays; an 17-Plex Milliplex MAP high-sensitivity human cytokine panel (Cat#HSTCMAG-28SK) for GM-CSF, IFNg, IL-1B, IL-2, IL-4, IL-5, IL-6, IL-7, IL-8, IL-10, IL-12(p70), IL-17A, IL-21, IL-23, ITAC, Fractalkine, and TNFa; and a 7-Pex Milliplex MAP human cytokine/chemokine panel (Cat#HCYTOMAG-60 K) for Eotaxin, IL-1a, IL-1RA, IL-15, IP-10, MDC, and MCP-1. Both assays were performed following kit instructions and read on a Luminex 200 instrument. Results were reported in pg/mL based on standard curve values.

### Statistical analysis

We grouped the study participant into two groups (responded and did not respond to Nystatin oral rinse) based on their salivary *C. albicans* status at the 1-week follow-up visit. The characteristics of the study participants from these two groups were compared using t-test for normal distributed continuous data (age, DFMT, and DMFS), Mann–Whitney U test for non-normal distributed data (weighted and non-weighted sweet/non-sweet indices), and chi-square or Fisher’s exact tests for categorical data including demographic characteristics (race, ethnicity, education level, marital status), medical background (hypertension, allergy to penicillin, long-term use of antibiotics, and smoking), oral health condition (tooth brushing frequency and use of nightguard), and detection of non-*albicans Candida species*. The Wilcoxon matched-pair signed-rank test was used to compare the changes of oral health conditions (BOP and PI) between the follow-up visits and baseline visit. Pair-wise t-test was used to compare the carriage of *S. mutans* (converted to natural log value), *C. albicans* (converted to natural log value), and salivary cytokines (converted to natural log value) between the follow-up visits and baseline visit. A multivariate logistic regression was used to analyze the factors (race, gender, ethnicity, age, educational level, hypertension, smoking, tooth brushing frequency, night guard usage, adherence to Nystatin rinse, weighted sweet index, and non-sweet index) associated with the response to Nystatin oral rinse. Response to oral rinse is defined as no detection of *C. albicans* in saliva at the 1-week follow-up visit. All statistical tests were two-sided with a significant level of 5%. SPSS IBM was used for statistical analyses.

## Results

The demographic-socioeconomic-medical-oral conditions information are shown in Table 1. A total of one hundred participants were screened, and twenty participants who met the inclusion and exclusion criteria advanced to the clinical trial. All study participants completed the baseline visits. Nineteen participants completed the 1-week and 3-month follow-up visits. One participant withdrew due to reported side effect of using Nystatin oral rinse and did not complete the 7-day rinse and the 1-week and 3-month follow-up visits. Based on whether *C. albicans* was detected at 1-week follow-up visit, we grouped the participants into did not respond group (*C. albicans* + at V2) and responded group (*C. albicans*– at V2). No statistical differences were seen between participants in these two groups, regarding age, gender, race, ethnicity, education level, employment status, marital status, medical conditions, smoking status, and oral health status (*p* > 0.05).

**Table 1:** Demographic-socioeconomic-medical-oral conditions and non-albicans *Candida* species

Category		All (*n* = 20)	*C.a* − at 1-week follow-up (*n* = 10)	*C.a* + at 1-week follow-up (*n* = 9)	*p*-value
*Demographic-socioeconomic*					
Age (year)		45.1 ± 11.6	43.1 ± 12.4	47.3 ± 10.6	0.40
Gender	Female Male	70% (14) 30% (6)	80.0% (8) 20.0% (2)	55.5% (5) 44.4% (4)	0.34
Race	White Black Other	40% (8) 10% (2) 50% (10)	40.0% (4) 0% (0) 60.0% (6)	44.4% (4) 11.1% (1) 44.4% (4)	0.84
Ethnicity	Non-Hispanic	90.0% (18)	90.0% (9)	100% (9)	0.47
Education	≤ High school College level > College level	20% (4) 25% (5) 55% (11)	30.0% (3) 30.0% (3) 40.0% (4)	11.1% (1) 22.2% (2) 66.6% (6)	0.13
Employed (Y)		75% (15)	60.0% (6)	88.8% (8)	0.30
Married (Y)	Married	65% (13)	50.0% (5)	77.7% (7)	0.35
*Medical condition*					
Hypertension		15% (3)	18.1% (2)	11.1% (1)	0.54
Allergy to penicillin		20% (4)	18.1% (2)	22.2% (2)	0.67
Long-term antibiotics		10% (2)	9% (1)	11.1% (1)	1.00
Smoking (Y)		20% (4)	27.2% (3)	11.1% (1)	0.58
Sweet index Sweet index (weighted)		1.7 ± 1.8 3.2 ± 4.2	0.9 ± 0.9 1.6 ± 2.3	2.7 ± 2.1 5.2 ± 5.3	0.37 0.60
Non-sweet index Non-sweet index (weighted)		3.2 ± 1.5 16.5 ± 10.4	2.4 ± 1.5 14.0 ± 13.4	4.1 ± 1.0 19.4 ± 3.7	0.82 0.94
*Oral health condition*					
Tooth brushing	Once daily ≥ Twice daily	25% (5) 75% (15)	27.2% (3) 72.7% (8)	22.2% (2) 77.7% (7)	0.58
Uses nightguard (Y)		15% (3)	9% (1)	22.2% (2)	0.58
DMFT (decayed, missing, filled teeth)		9.0 ± 5.8 0.9 ± 1.2 1.1 ± 1.4 7.0 ± 5.1	8.8 ± 5.2 0.6 ± 0.9 1.3 ± 1.4 6.7 ± 4.5	9.2 ± 6.8 1.2 ± 1.4 0.7 ± 1.3 7.2 ± 5.9	0.71
DT					0.37
MT					0.52
FT					0.71
DMFS (decayed, missing, filled surface)		26.7 ± 22.6 1.9 ± 3.7 4.8 ± 6.4 19.6 ± 16.7	25.1 ± 20.0 1.0 ± 1.8 5.0 ± 5.9 19.1 ± 14.7	28.5 ± 26.4 2.8 ± 5.1 4.4 ± 7.2 20.1 ± 19.7	0.64
DS					0.32
MS					0.88
FS					0.83
*Detection of non-albicans Candida species*					
*C. krusei* (saliva)	Baseline	10% (2)	20% (2)	0% (0)	0.48
	1-week follow-up	0% (0)	0% (0)	0% (0)	NA
	3-month follow-up	5% (1)	10% (1)	0% (0)	1.00
*C. krusei* (plaque)	Baseline	0% (0)	0% (0)	0% (0)	NA
	1-week follow-up	0% (0)	0% (0)	0% (0)	NA
	3-month follow up	0% (0)	0% (0)	0% (0)	NA
*C. glabrata* (saliva)	Baseline	20% (4)	30.% (3)	11% (1)	1.00
	1-week follow-up	15% (3)	20% (2)	11% (1)	1.00
	3-month follow-up	20% (4)	30% (3)	11% (1)	0.94
*C. gla*brata (plaque)	Baseline	5% (1)	10% (1)	0% (0)	1.00
	1-week follow-up	5% (1)	10% (1)	0% (0)	1.00
	3-month follow-up	0% (0)	0% (0)	0% (0)	NA

### Adherence to Nystatin rinse

Participants were instructed to rinse with 6 mm of 100,000 IU/ml Nystatin oral suspension, four times per day for 7 days, with a total of 28 applications. The mean number of medication usage among participants was 24.4 ± 4.8 doses, where 8 participants completed all doses as instructed, 5 missed less than 10% of the doses, 5 missed 11–20%, and 3 participants missed 40–60% of the doses. Only one participant complained of mild side effects that cleared up shortly (nausea and sore throat) after taking the third dose on the third day. The participant was instructed to stop taking the medication, and a follow-up appointment was scheduled for further evaluation.

### Oral *C. albicans* carriage following Nystatin oral rinse

**Fig. 2:**
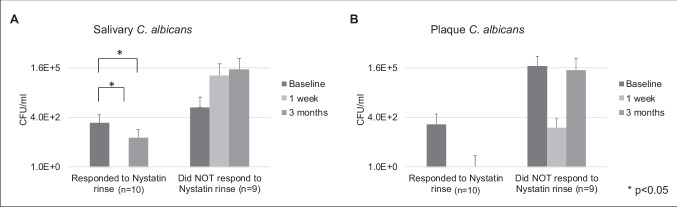
Oral *Candida* carriage following Nystatin oral rinse. **A** Carriage of salivary *C. albicans*. Total elimination of salivary *C. albicans* at the 1-week follow-up and a lightly bounced back salivary among 10 participants. The participants who responded to Nystatin. The reduction of salivary *C. albicans* between the baseline and follow-up visits were statistically significant (*p* < 0.05). **B** Carriage of plaque *C. albicans*. Reduction of plaque *C. albicans* were seen following Nystatin rinse among the same participants; however, no statistical differences were detected between the follow-up and baseline visits (*p* > 0.05)

The changes of salivary and plaque *C. albicans* are shown in Fig. 2. The participants who responded to Nystatin oral rinse had a salivary carriage of *C. albicans* of 2.0 ± 3.6 × 10^2^ CFU/ml at the baseline, a total elimination of salivary *C. albicans* at the 1-week follow up, and a lightly bounced back salivary *C. albicans carriage* to 3.4 ± 6.0 × 10^2^ CFU/ml at 3-month follow-up. The reduction of salivary *C. albicans* between the baseline and follow-up visits was statistically significant (*p* < 0.05) (Fig. 2A). Reduction of plaque *C. albicans* was seen following Nystatin rinse among the same participants; however, no statistical differences were detected between the follow-up and baseline visits (*p* > 0.05).

The participants who did not respond to Nystatin oral rinse had a salivary carriage of *C. albicans* of 1.4 ± 3.2 × 10^3^ CFU/ml at baseline visit and 6.4 ± 19 × 10^4^ and 1.3 ± 3.7 × 10^2^ CFU/ml at 1-week and 3-month follow-ups, respectively (Fig. 2A). Plaque *C. albicans* carriage was 2.0 ± 4.1 × 10^5^ CFU/ml at baseline, decreased to 1.2 ± 2.3 × 10^2^ CFU/ml at 1-week follow-up, and bounced back to 1.2 ± 3.6 × 10^5^ CFU/ml at 3-month follow-up (Fig. 2B).

Interestingly, we found that the responses to Nystatin oral rinse among the study participants was not associated with any of the independent variables tested, such as race, gender, age group, oral hygiene practice, adherence to Nystatin rinse, or sweet consumption (*p* > 0.05).

### Oral *S. mutans* carriage following Nystatin oral rinse

**Fig. 3:**
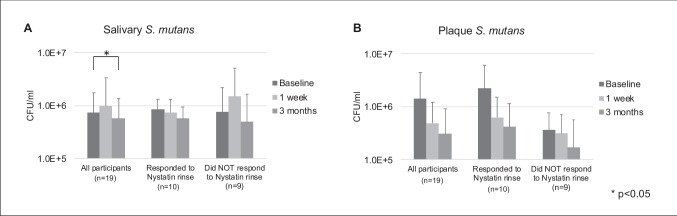
Oral *S. mutans* carriage following Nystatin oral rinse. **A** Carriage of salivary *S. mutans*. A significant overall reduction of salivary *S. mutans* was observed in V3 comparing to the baseline visit (*p* < 0.05). **B** Carriage of plaque *S. mutans*. Overall reduction of plaque *S. mutans* were seen following treatment. However, no statistical differences were detected between the follow-up and baseline visits (*p* > 0.05)

The changes of salivary and plaque *S. mutans* are shown in Fig. 3. We observed an overall salivary *S. mutans* carriage of 7.5 ± 10.1 × 10^5^ CFU/ml at the baseline and a significantly decrease to 5.7 ± 8.0 × 10^5^ CFU/ml at 3-month follow-up (*p* < 0.05) (Fig. 3A).

Among participants who responded to Nystatin rinse, a reduction in salivary *S. mutans* was observed in 1-week and 3-month follow-ups comparing to baseline 8.5 ± 4.7 × 10^5^ CFU/ml, 7.5 ± 5.7 × 10^5^ CFU/ml, and 5.7 ± 3.6 × 10^5^ CFU/ml, respectively. However, no statistical differences were detected between the follow-up and baseline visits in both groups (*p* > 0.05) (Fig. 3A). Similar trend of *S. mutans* reduction in plaque samples was also observed among participants who responded to Nystatin rinse; however, no statistical significance was found (*p* > 0.05).

The participants who did not responded to Nystatin oral rinse had a carriage of 3.7 ± 4.1 × 10^5^ CFU/ml *S. mutans* in plaque samples at the baseline and a slight reduction of *S. mutans* carriage to 1.7 ± 4.1 × 10^5^ CFU/ml at 3-month follow-up. However, changes were not statistically significant (*p* > 0.05) (Fig. 3B).

### Periodontal status following Nystatin oral rinse

**Fig. 4:**
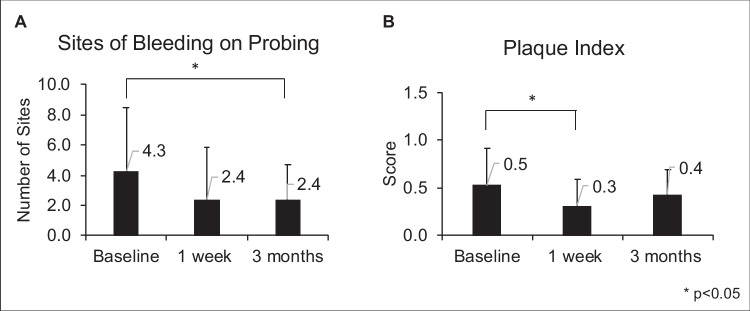
Bleeding on probing and plaque index following Nystatin oral rinse. **A** Bleeding on probing (BOP) in number of interproximal sites. Significant improvement in BOP following treatment observed (*p* < 0.05). **B** Plaque index (PI). Plaque index score significantly improved following treatment at V2

We used BOP and PI to assess the periodontal status and plaque control of participants. The changes of BOP sites and plaque index are shown in Fig. 4. At the baseline visit, the number of BOP sites was 4.3 ± 4.2. The BOP sites were reduced to 2.4 ± 3.5 at the 1-week follow-up and to 2.4 ± 2.3 at the 3-month follow-up (*p* < 0.05) (Fig. 4A). The plaque accumulating measured by PI was also significantly reduced (Fig. 4B), with a statistical significance between the baseline and 1-week follow-up visit (*p* < 0.05).

### Susceptibility of* C. albicans *clinical isolates to Nystatin in vitro

No isolates of *C. albicans* were resistant to in vitro Nystatin susceptibility tests. The value of MIC50 ranges from 0.03 to 0.06 ug/ml, and the value of MIC90 ranges from 0.06 to 0.23 ug/ml.

### Changes of salivary cytokines following Nystatin oral rinse

**Fig. 5:**
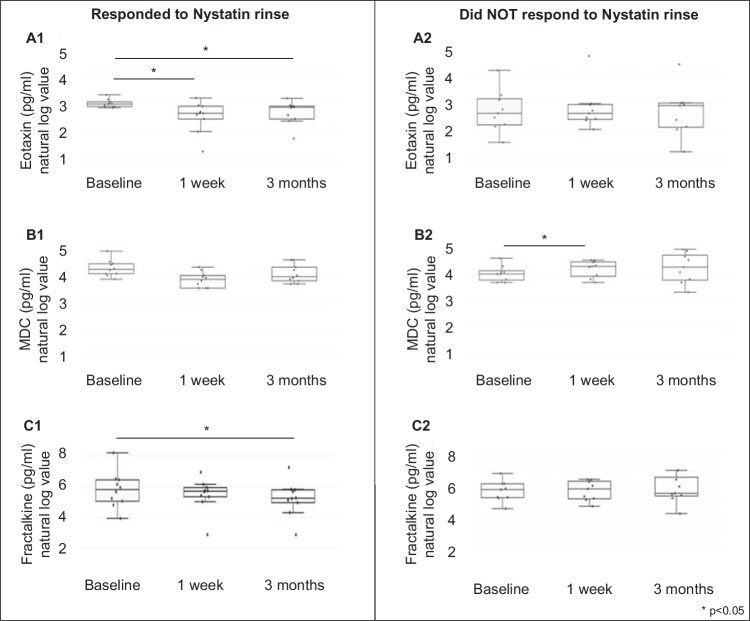
Changes of salivary immune markers following Nystatin oral rinse. (A) Salivary level of eotaxin among participants who responded to Nystatin rinse (A-1) and those who did not respond to Nystatin rinse (A-2). (B) Salivary level of macrophage-derived chemokine (MDC) among participants who responded to Nystatin rinse (B-1) and those who did not respond to Nystatin rinse (B-2). (C) Salivary level of fractalkine among participants who responded to Nystatin rinse (C-1) and those who did not respond to Nystatin rinse (C-2). The salivary immune marker (pg/ml) concentration was converted to natural log value (Ln). The values of Ln 1–8 equal to 2.7, 7.4, 20.0, 54.6, 148.4, 403.4, 1096.6, and 2981.0

Among 24 cytokines assessed in saliva, 13 were detected with valid value across the study participants. We found that the levels of Eotaxin and Fractalkine were significantly reduced at 3-month follow-up among participants who responded to Nystatin rinse (*p* < 0.05) (see Fig. 5A-1 and C-1). Worth noting, the changes were not seen among the individuals who did not respond to Nystatin rinse (Fig. 5A-2 and C-2). In contrast, salivary level of macrophage-derived chemokine (MDC) among participants who did not respond to Nystatin rinse had an elevation at 1-week follow-up compared to the baseline visit (Fig. 5B-2). No statistical differences were detected among other salivary cytokines (values of 13 cytokines; see Table S1).

## Discussion

### *Candida* carriage and diagnostic criteria

*Candida* species frequently present in the normal flora of the oral cavity without causing clinical symptoms of candidiasis [31]. The oral *Candida* carriage ranges from 20 to 75% in the general population but is typically around 45% in the neonates, 45–65% in healthy children, 30–45% in healthy adults, and 50–65% in adults wearing removable dentures [32–36]. Oral *Candida* becomes pathogenic when the oral flora change occurs under the change of specific host factors [37]. These factors include impaired salivary gland function, denture wearing, long-term use of broad-spectrum antibiotics, corticosteroids and antidepressants, patients with diabetes mellitus, renal failure, hyperthyroidism, cancer, and HIV infection [38–40].

Oral candidiasis is often diagnosed upon patient-reported symptoms and clinical manifestations, such as a white coating on the tongue and white or red patches on the cheek mucosa. Oral candidiasis could also be diagnosed based on the laboratory assay [41]. One of the laboratory criteria for diagnosing oral candidiasis is the presence of ≥ 400 colony forming units (CFU)/mL of salivary *Candida*, even without clinical symptoms [25].

### Factors associated with recurrent oral Candidiasis and failed response to Nystatin rinse

#### Host factors

It has become evident that virulence factors are associated with the shift of *C. albicans* from being harmless commensal to pathogenic organism inside the oral cavity [42]. Besides, other host-related factors, such as compromised autoimmunity, tobacco consumption, hyposalivation, denture wearing, systemic disease, and medication, were found critical in developing oral candidiasis [43]. Therefore, the outcomes of clinical interventions may differ depending on these host-related factors and their severity. For example, the emergence of antifungal resistance is observed more frequently among HIV patients receiving long-term azole therapy [44]. Under the circumstance that host factors are unable to modify, such as administration of antifungal treatment among immunocompromised patients and patients with malignancies, oral candidiasis could be intervened by administering systemic antifungal agents, followed by prophylactic antifungal treatment [45].

#### Microbial factors

Another phenomena associated with response to antifungal treatment is the presence of non-albicans species, particularly *C. glabrata* and *C. parapsilosis*; these species could contribute to the onset of oral candidiasis and the development of resistance against antifungal medications [46]. Furthermore, the resistance of *C. Krusei* and *C. glabrata* against fluconazole has been reported [47]. As a result, a new class of antifungal agents, Echinocandin, has been designed to overcome the issue of fungal resistance [48]. Thus, determining the *Candida* species that are responsible for the disease via culturing and drug-susceptibility tests is critical for successful antifungal treatment.

#### Medication factors

In the 1950s, Nystatin and amphotericin B were first invented to treat oral candidiasis. They are supplied in different forms, including pastilles, lozenges, suspension, troches, suppositories, and coated tablets. However, utilizing the appropriate form of Nystatin and amphotericin B is essential to obtain the optimum outcomes. As these two agents cannot be absorbed from the gastrointestinal tract, it is not advised to swallow them orally as tablets [49]. Instead, sucking and dissolving these agents inside the oral cavity could effectively treat oral candidiasis. This approach allows topical application of the agent, where the agent is applied to the infected area. Doubling the application time of Nystatin or topical amphotericin B has been suggested to improve the clinical efficiency of the treatment. However, prolonging the treatment duration may not be convenient for the patient due to taste intolerance, which may compromise the patients’ compliance [50]. Therefore, the oral care provider should instruct the patient to use the treatment properly to prevent infection recurrence.

### *S. mutans* reduction following Nystatin rinse

Our study suggested that the *S*. *mutans* carriage was reduced following Nystatin treatment. Several possible mechanisms could explain this reduction. Many studies show that the coexistence of *C. albicans* and *S. mutans* may increase biofilm (plaque) virulence and intensify the severity of the disease [17, 21, 51]. For instance, faster onset of disease, higher number, and more severe smooth-surface lesions were observed with *C. albicans* with *S. mutans* growing together in the presence of sucrose compared to infection with either organism alone in an animal model [17]. The associated pathogenicity appears to be related to the *S*. *mutans*-derived exoenzyme glucosyltransferase B (GtfB), a key exopolysaccharide (EPS) producer. GtfB can bind to the surface of *C*. *albicans* cells in an active form and produce EPS locally that provide enhanced binding sites for *S*. *mutans*, which in turn promote the formation of biofilms containing elevated EPS amounts and high numbers of *S*. *mutans* and *C*. *albicans* [17, 18, 52]*.*

### Association between Candida and periodontal disease

Several studies revealed an association between *Candida* species and periodontal disease, for instance, an increased number of *Candida* species, particularly *C. albicans*, in patients with chronic periodontitis patients, when comparing to patients with healthy periodontium [53]. Although the exact pathogenic mechanism by which *Candida* contribute to periodontal disease progression remains unclear, *Candida* virulence factors such as adhesion, invasion, dimorphism, and biofilm formation of *Candida* have been suggested involved in the pathogenesis [54]. One of the main proposed pathogenic mechanisms is that *C. albicans* may have a role in periodontal microbial plaque infrastructure and its adherence to the periodontal tissues [55]. *Candida* was typically found in the outer layers of the plaque and appeared to act as a barrier between the host immunity and the inner layers of the mixed biofilm [55]. Therefore, *C. albicans* could have a role in the immune evasion of the plaque in periodontal infections and in stimulating destructive inflammation in the underlying tissues. In our study, the improved periodontal parameters such as plaque index and bleeding index and reduced plaque accumulation among study participants could be due to the phenomena described above. However, since this study did not assess the periodontal pocket depth, we are not able to comprehensively assess the changes in the periodontal status of the study participants.

### Salivary immune marker changes following Nystatin rinse

Interestingly, our results revealed a reduction of salivary cytokine/chemokine eotaxin among participants who responded to oral rinse and did not have *C. albicans* at 1-week and 3-month follow-ups. Chemokines are secretory proteins involved in several biological functions, such as direct migration of leukocytes, activation of immune responses, tumor behavior modulation through tumor-associated angiogenesis, tumor cell proliferation, and innate and adaptive host responses [56]. Human eotaxin is a 74 amino acid polypeptide, non-glycosylated protein chemokine, expressed by various cells, including endothelial cells, epithelial cells, broncho-alveolar macrophages, lung and dermal fibroblasts, smooth muscle cells, and chondrocytes [57]. Eotaxins bind to the CCR3 receptor (CC chemokine receptor 3) and selectively recruit eosinophils [58]. In addition to recruiting, it aggregates eosinophils and is also a chemoattractant for eosinophils, basophils, helper T cells, macrophages, and mast cells. Increased circulating eosinophils are pathognomonic characteristics for several allergic and inflammatory disorders and malignancies. Eotaxins can stimulate the migration of eosinophils from blood plasma to tissues through CCR3 activation [58]. As a result, they are implicated in the pathogenesis of several allergic conditions (atopic dermatitis, allergic rhinitis, and asthma), inflammatory diseases (inflammatory bowel disease, ulcerative colitis, Crohn’s disease, eosinophilic gastroenteritis, chronic sinusitis, nasal polyposis), as well as malignancies (Hodgkin’s lymphoma and leukemia) [58]. Studies have reported using salivary proteins as biomarkers that could help classify caries susceptibility in an individual. Paque and colleagues analyzed saliva samples from healthy/gingivitis, caries-affected/ gingivitis, and caries-affected/healthy patients for cytokines, chemokines, growth factors, and proteolytic enzymes as oral microbiomes. Four potential salivary biomarkers were identified (IL-4, 13, 2-ra, and chemokine eotaxin/CCl11) as discriminatory and could be used to determine the caries susceptibility of individuals. All four biomarkers for caries-affected patients were observed, including chemokine eotaxin (1.7 pg/mL ± 0.9 in a patient with deep caries) [59]. Reduced status of eotaxin among participants who were free of *Candida* might indicate an altered immune status and reduced risk for caries.

### Study limitation

The following limitations need to be considered when interpreting our study results: (1) single-arm study with no control group, (2) limited sample size, and (3) the study was only conducted in one US city. Thus, generalization to other populations is unreliable.

### Future direction and next step

Microbiome study to assess the effect on oral microbial changes.

## Conclusions

Our study results indicate that oral antifungal treatment may positively affect the carriage of oral cariogenic microorganisms, dental plaque formation, and periodontal status evaluated by bleeding on probing. However, future clinical trials are warranted to comprehensively assess the impact of antifungal treatment on the oral flora other than *S. mutans* and Candida.

## Supplementary information

Below is the link to the electronic supplementary material.

Supplementary file1 (DOCX 18 KB)

## Data Availability

The data presented in this study are included in the manuscript and supplement table.
