# Supplementary material for: Effects of Nystatin oral rinse on oral Candida species and Streptococcus mutans among healthy adults
Source: Clin Oral Investig. 2023 Mar 24;27(7):3557–68. doi: 10.1007/s00784-023-04969-5 (PMC10329597; doi:10.1007/s00784-023-04969-5)
Supplement: Supplementary file 1 — Supplementary file1 (DOCX 18 KB) [file 784_2023_4969_MOESM1_ESM.docx]

| Groups | VISIT | | Eotaxin | MDC | IL-15 | IL-1RA | IL-1a | IP-10 | MCP-1 | TNFa | Fractalkine | GM-CSF | IL-10 | IL-1B | IL-8 |
| --- | --- | --- | --- | --- | --- | --- | --- | --- | --- | --- | --- | --- | --- | --- | --- |
| Responded to Nystatin rinse (n=10) | Baseline | Mean | 19.00 | 78.70 | 4.86 | 37722.31 | 2591.63 | 192.00 | 892.90 | 28.65 | 667.02 | 8.62 | 7.42 | 250.96 | 1281.52 |
|  |  | SD | 2.77 | 27.39 | 2.26 | 12093.15 | 1520.14 | 121.89 | 603.17 | 17.60 | 1084.33 | 6.07 | 10.48 | 372.45 | 918.64 |
|  | 1 week | Mean | 13.36 | 50.40 | 3.62 | 39512.63 | 2013.40 | 208.89 | 748.50 | 22.72 | 343.74 | 8.20 | 6.10 | 77.04 | 958.40 |
|  |  | SD | 5.36 | 14.85 | 2.22 | 4831.63 | 1235.70 | 146.80 | 389.66 | 15.91 | 276.83 | 5.36 | 8.79 | 68.05 | 531.34 |
|  | 3 months | Mean | 14.71 | 64.07 | 5.25 | 39657.00 | 2307.64 | 161.79 | 963.59 | 28.79 | 317.16 | 7.21 | 3.86 | 109.72 | 1029.31 |
|  |  | SD | 4.74 | 23.69 | 2.77 | 5151.37 | 1186.63 | 116.38 | 570.34 | 22.48 | 398.68 | 4.55 | 4.30 | 102.72 | 1037.26 |
| Did NOT respond to Nystatin rinse (n=9) | Baseline | Mean | 19.93 | 60.42 | 4.47 | 33616.95 | 2084.53 | 627.67 | 840.64 | 23.84 | 382.67 | 8.64 | 14.06 | 179.28 | 1136.67 |
|  |  | SD | 17.91 | 19.44 | 2.07 | 13773.08 | 1485.98 | 482.33 | 344.26 | 30.96 | 257.06 | 4.85 | 17.48 | 246.91 | 1323.38 |
|  | 1 week | Mean | 24.22 | 72.04 | 5.41 | 30866.46 | 1375.17 | 1279.35 | 985.58 | 16.63 | 382.81 | 13.00 | 36.52 | 126.00 | 926.76 |
|  |  | SD | 30.99 | 20.27 | 2.61 | 13411.77 | 1017.51 | 1488.72 | 522.51 | 20.28 | 210.33 | 14.08 | 96.61 | 240.59 | 1205.94 |
|  | 3 months | Mean | 21.00 | 82.14 | 5.39 | 34725.63 | 2036.21 | 727.70 | 971.56 | 110.02 | 977.58 | 10.41 | 34.47 | 500.08 | 1339.43 |
|  |  | SD | 22.63 | 42.11 | 3.17 | 12037.83 | 1811.83 | 747.92 | 684.78 | 232.13 | 1711.10 | 10.84 | 69.38 | 1336.41 | 1843.00 |

**Table S1 Salivary cytokines among participants (unit: pg/ml)**
